# Optimizing Sowing and Flooding Depth for Anaerobic Germination-Tolerant Genotypes to Enhance Crop Establishment, Early Growth, and Weed Management in Dry-Seeded Rice (*Oryza sativa* L.)

**DOI:** 10.3389/fpls.2018.01654

**Published:** 2018-11-23

**Authors:** Buddhika Sampath Chamara, Buddhi Marambe, Virender Kumar, Abdelbagi M. Ismail, Endang M. Septiningsih, Bhagirath Singh Chauhan

**Affiliations:** ^1^Crop and Environmental Sciences Division, International Rice Research Institute, Los Baños, Philippines; ^2^Postgraduate Institute of Agriculture, University of Peradeniya, Peradeniya, Sri Lanka; ^3^Department of Crop Science, Faculty of Agriculture, University of Peradeniya, Peradeniya, Sri Lanka; ^4^International Rice Research Institute, Los Baños, Philippines; ^5^Department of Soil and Crop Sciences, Texas A&M University, College Station, TX, United States; ^6^The Centre for Crop Science, Queensland Alliance for Agriculture and Food Innovation, The University of Queensland, Gatton, QLD, Australia

**Keywords:** AG tolerance, direct seeding, weed competition, *Echinochloa crus-galli*, *Ludwigia hyssopifolia*, *Cyperus difformis*

## Abstract

Poor crop establishment, high weed infestation, and consequent yield loss are major concerns for dry-seeded rice (DSR). Flooding after seeding helps in managing weeds but reduces seed germination and crop stand. Anaerobic germination (AG)-tolerant rice genotypes could overcome these problems in DSR. Screenhouse experiments were established to evaluate the effect of seed sowing depth (SD) (0.5 cm, 1 cm, and 2 cm) and flooding depth (FD) (saturated, 2 cm, and 5 cm) on crop establishment, early growth, and weed competitiveness in DSR using AG-tolerant genotypes (Khao Hlan On, Ma-Zhan Red, IR64+AG1, and IR64). *Echinochloa crus-galli*, *Ludwigia hyssopifolia*, and *Cyperus difformis* were used in the weedy treatment. Rice plants reached maximum emergence 9–13 days later under flooding compared with saturated conditions. Crop emergence decreased by 12–22% at 0.5 and 1 cm SD and by 48–60% at 2 cm SD, when combined with 2 or 5 cm FD compared with saturated conditions. The 2 cm SD reduced seedling emergence by 23–42% in Khao Hlan On and Ma-Zhan Red, by 62–70% in IR64+AG1, and by 90–92% in IR64 under flooding. Initial growth in rice plant height was slow under flooding but increased progressively after the seedlings emerged from water and the final height was not affected by FD. Leaf area, total shoot biomass, tiller density, and leaf number per pot of rice were higher at 1 cm SD (*P*< 0.05), but decreased drastically at 2 cm SD under flooding. The emergence of *E. crus-galli* and *L. hyssopifolia* decreased by 53–65% and 89–95%, respectively, but increased by 49–68% in *C. difformis* under 2 and 5 cm FD, respectively, compared with saturated conditions. The shoot biomass of the weeds followed the same trend. Khao Hlan On showed the highest weed-competitive ability under all FD while the biomass of IR64+AG1 and IR64 decreased by 10–14% due to weed competition under 2 cm FD. The 1 cm SD showed better growth for all genotypes under different FD. The 2 cm FD is sufficient to have a significant control of problematic weed species. The tolerance of AG of rice genotypes should be further enhanced to increase their weed-competitive ability.

## Introduction

Global food security is being threatened by the aggravating effects of climate change (Schmidhuber and Tubiello, 2007; Furuya and Kobayashi, 2009). Cereal grains play a crucial role in global food security, accounting for more than 50% of the world’s daily caloric intake. Rice is the second-largest cereal crop in the world and more than 90% of world rice production comes from Asia (Awika, 2011). Rice consumes more water than other cereals, mainly because of the higher water requirement for land preparation of transplanted rice and higher water loss associated with continuous flooding of paddy fields, through evapotranspiration, seepage, and percolation (Renault, 2004). However, climate change is affecting rainfall intensity and distribution, and competition for water from non-agricultural activities is growing, causing progressive scarcity in water resources for agricultural use (Morrison et al., 2009; Turral et al., 2011). This has been one of the main reasons for the current trend of switching from puddled transplanted rice (PTR) to dry-seeded rice (DSR). DSR has been long recognized as a more resource- and energy-efficient and climate-resilient rice crop establishment method (Rao et al., 2007; Chauhan and Johnson, 2010). Labor scarcity and the high production cost of PTR are the other two major drivers of this shift (Kumar and Ladha, 2011; Matloob et al., 2015). In addition, DSR has a higher potential of mitigating greenhouse gas emissions than PTR, thereby reducing global warming (Kumar and Ladha, 2011; Bhullar et al., 2016; Tao et al., 2016; Tirol-Padre et al., 2016).

High weed infestation and consequently higher yield losses are some of the major risks associated with DSR (Rao et al., 2007; Kumar and Ladha, 2011; Singh et al., 2011; Chauhan, 2012; Jabran and Chauhan, 2015). The yield loss in DSR due to weed competition has been estimated at 15% (Baltazar and De Datta, 1992) to 40% (Herath Banda et al., 1998), and could be as high as 85–100% in the absence of weed control measures (Rao et al., 2007; Singh et al., 2011; Mahajan and Chauhan, 2013). This is mainly due to the simultaneous emergence of weeds with rice and the absence of the weed-suppressive effect of puddling and flooding in the early stages of crop growth in the DSR system (Rao et al., 2007; Singh et al., 2007; Chauhan, 2012; Kumar et al., 2013). This has resulted in increased dependence on herbicides for weed control in DSR systems, which, in some cases, might not provide sustainable and season-long weed control when used alone (Pacanoski and Glatkova, 2009; Chauhan et al., 2015). Therefore, IWM strategies are critical in establishing sustained weed control in DSR systems.

Poor and uneven crop establishment is another risk associated with DSR attributed to flooding/waterlogging immediately after seeding or during early seedling growth of rice caused by unpredictable heavy monsoon rainfall, poor leveling of fields, and poor drainage (Konchan and Kono, 1996; Chauhan, 2012; Ismail et al., 2012), which will consequently lead to reduced weed competitiveness. In addition, water management is the most important IWM strategy in DSR (Caton et al., 2002). Flooding the field following direct seeding creates anaerobic conditions, which help manage weeds in the early stages of crop growth (Ampong-Nyarko and De-Datta, 1991; Rao et al., 2007), but also reduces germination of rice as modern rice genotypes are sensitive to flooded/anaerobic conditions during germination and early seedling growth (Das et al., 2009; Ismail et al., 2009). The increasing trends of adoption of DSR therefore demand improved crop establishment and weed management techniques in both irrigated and rainfed ecosystems (Konchan and Kono, 1996). Rice genotypes that are tolerant of anaerobic conditions during germination and early seedling growth would overcome these problems, making DSR more attractive to farmers by enhancing crop establishment in flooded soils and facilitating weed management through flooding (Ismail et al., 2009, 2012; Mahajan and Chauhan, 2013).

After screening more than 8000 GenBank accessions, a few landraces with tolerance of AG were identified, including Khao Hlan On, Ma-Zhan Red, Khaiyan, Kalongchi, and Nanhi (Angaji et al., 2010; Septiningsih et al., 2013b). Several promising QTLs for AG tolerance were identified, including *qAG-9-2* (AG1) and *qAG7.1* (AG2) derived from Khao Hlan On and Ma-Zhan Red, respectively. Using marker-assisted backcrossing (MABC), these QTLs were introgressed in the backgrounds of high-yielding rice varieties IR64, PSB Rc82, and Ciherang (Collard et al., 2013; Kretzschmar et al., 2015; Toledo et al., 2015; Septiningsih and Mackill, 2018). Although basic agronomic information on rice crop establishment using DSR is fairly available when rice is grown under aerobic conditions (Kumar and Ladha, 2011; Matloob et al., 2015), such information is not available for rice sown under anaerobic or flooded soil conditions as in lowlands. Deeper sowing in saturated and/or flooded soils typical of lowland conditions will reduce crop establishment and early seedling growth. In addition, the optimum seeding density for variable SD and FD has not yet been established for the new AG-tolerant rice varieties recently becoming available. Optimizing these agronomic practices is important for achieving better crop establishment and weed control in DSR when water is efficiently used with AG-tolerant genotypes. This study, therefore, attempted to establish the optimum seed SD and FD for these new genotypes and assess their effects on crop establishment and weed management in DSR.

## Materials and Methods

### Experimental Site

**FIGURE 1:**
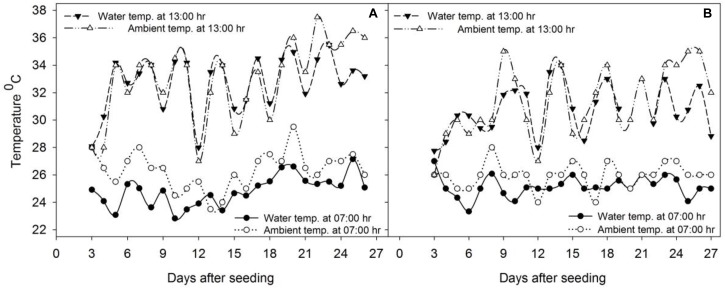
Ambient and mean water temperature (°C) during the study period of **(A)** experiment 1 and **(B)** experiment 2 measured at 07:00 and 13:00 h.

The experiments were conducted in a screenhouse (a steel-framed large chamber confined with 2 mm steel mesh with ecological conditions comparable with those of the field) at the International Rice Research Institute (IRRI), Philippines, during 2014. The study consisted of two experiments, each repeated twice. The ambient and water temperature during the study period is shown in Figure 1.

### Plant Materials

Two AG-tolerant accessions, Ma-Zhan Red from China and Khao Hlan On from Myanmar (*Oryza sativa* L. subsp. *japonica*), AG-tolerant rice breeding line IR64+AG1 recently developed through introgression of *AG1* (a major QTL responsible for AG tolerance derived from Khao Hlan On; Angaji et al., 2010) into the moderately tolerant rice genotype IR64 (subsp. *indica*), and the recurrent parent IR64 were used in this study (Datta and Datta, 2006; Angaji et al., 2010). These genotypes were directly sown on the IRRI experimental farm under control conditions during the wet season of 2013 and the harvested seeds were used in this study. Three major weed species common in DSR, including a grass [*Echinochloa crus-galli* (L.) P. Beauv.], a broadleaf [*Ludwigia hyssopifolia* (G. Don) Exell], and a sedge (*Cyperus difformis* L.), were included to assess the level of suppression by flooding and weed competitiveness of the AG-tolerant genotypes. Seeds of these weed species were collected from several rice fields within IRRI’s experimental farm.

### Experimental Design and Data Collection

The experiments were established in a split–split plot arrangement within a randomized complete block design with three replicates (Supplementary Figure S1). In the first study; three FDs, 0 cm (saturated), 2 cm, and 5 cm, were the main plot factor; three SDs, 0.5 cm, 1 cm, and 2 cm, were the subplots; and four rice genotypes, Khao Hlan On, Ma-Zhan Red, IR64+AG1, and IR64, were the sub-subplot factor. Plastic pots (24 cm in diameter and 24 cm in height) were filled with non-sterilized soil (normal upland soil) up to 13 cm from the bottom, followed by a 5 cm sterilized soil layer (free from viable weed seeds) on top. The topsoil layer was filled in two steps based on the respective SDs. In the first step, the sterilized soil was added up to 4.5 cm, 4 cm, and 3 cm heights in pots assigned for SDs of 0.5 cm (SD0.5), 1 cm (SD1), and 2 cm (SD2), respectively. This was followed by seeding and then covering the seeds with 0.5 cm, 1 cm, and 2 cm of sterilized soil. The pots assigned for saturated conditions were pierced with a 1 cm diameter hole at 5 cm height from the bottom of the pots. Thirty rice seeds were sown equidistantly in each pot equivalent to a seeding rate of 150 kg ha^−1^. Equal distance between plants was approximated by sowing the seeds in five lines in each pot, that is, eight seeds in the middle line, followed by two lines with six seeds each and the last two outside lines with five seeds each (5—6—8—6—5). The FDs (2 cm and 5 cm) and saturated conditions (control) were imposed immediately after seeding. Saturated pots were irrigated at regular intervals to keep the soil moist. The flooding conditions were maintained until 28 DAS and then maintained saturated until 42 DAS. In order to facilitate two destructive samplings at 21 and 42 DAS, two sets from each experimental unit was maintained where one pot considered as one experimental unit (FD, SD, and CV combination). At each destructive sampling time, the whole experimental unit was harvested.

The second study was conducted to evaluate the weed competitiveness of three rice genotypes, which were selected based on their performance in the first study. The main plot consisted of three FDs, 0 cm (saturated), 2 cm, and 5 cm; the subplots were two weed levels, weedy (three weed species) and weed-free; and the sub-subplots consisted of three rice genotypes (Khao Hlan On, IR64+AG1, and IR64). Thirty rice seeds were sown at 1 cm depth as in the first study. Fifty seeds of each weed species were uniformly mixed in the uppermost 0.5 cm soil layer of each weedy pot, followed by leveling. Flooding and saturated treatment conditions were introduced immediately after seeding. The experiment was maintained for 42 DAS. Nitrogen in the form of urea was added in both studies in two equal splits at 14 and 28 DAS at the rate of 50 kg ha^−1^ in each split.

Seedling emergence, height, and leaf number of the rice plants were measured at weekly intervals. The tiller density, leaf area, and shoot biomass of the rice genotypes were measured at 21 and 42 DAS. Emerging weed seedlings were counted weekly and biomass of their shoots and roots was measured at 42 DAS. Plant height was measured from the soil surface to the tip of the longest leaf. The plants were uprooted and washed gently to remove soil particles adhering to the roots. Leaf area was measured using a leaf area meter (LI-3100C, United States), and the biomass of both rice and weeds was measured after drying in an oven at 70°C for 120 h. The biomass was then weighed using an analytical balance (AG204, Mettler Toledo, Switzerland).

### Statistical Analysis

For both studies, data were pooled over the experimental runs as there was no treatment by experimental run interaction. The pooled data were subjected to analysis of variance (ANOVA) (GenStat for Windows 17th Edition). Means were compared using Fisher’s protected LSD test at α = 0.05. Regression analysis was performed when ANOVA indicated a significant *F*-value for the tested factor. The sigmoid or exponential function was fitted to the data using regression analysis (SigmaPlot version 12.5, Systat Software, Inc., San Jose, CA, United States). Seedling emergence percentage, plant height, and leaf number per plant of rice were fitted to a three-parameter sigmoid function (Equation 1):

(1) $$y=a/(1+e^{\left[-(x-x0)/b\right]})$$

where *y* = estimated emergence percentage, plant height, or leaf number per plant; *x* = time in DAS; *a* = the maximum value of the test parameter; *x*_0_ = time to reach 50% of the final value of the test parameter; and *b* = slope. The % rice emergence at 35 DAS was fitted to a three-parameter exponential decay model (Equation 2):

(2) $$y=y_{0}+{\mathrm{ae}}^{\left(-\mathrm{bx}\right)}$$

where *y* = estimated emergence percentage, *y*_0_ = the baseline parameter, *x* = flooding depth, *a* = *y*-intercept, and *b* = slope. The relationships of rice leaf area, TSBM, tiller density per pot, and emergence percentage of the weeds were described using a two-parameter exponential decay model (Equation 3):

(3) $$y={\mathrm{ae}}^{\left(-\mathrm{bx}\right)}$$

where *y* = estimated rice leaf area, TSBM, tiller density per pot, and emergence percentage of the weeds; *x* = flooding depth; *a* = intercept; and *b* = slope.

## Results and Discussion

### Rice Seedling Emergence

**FIGURE 2:**
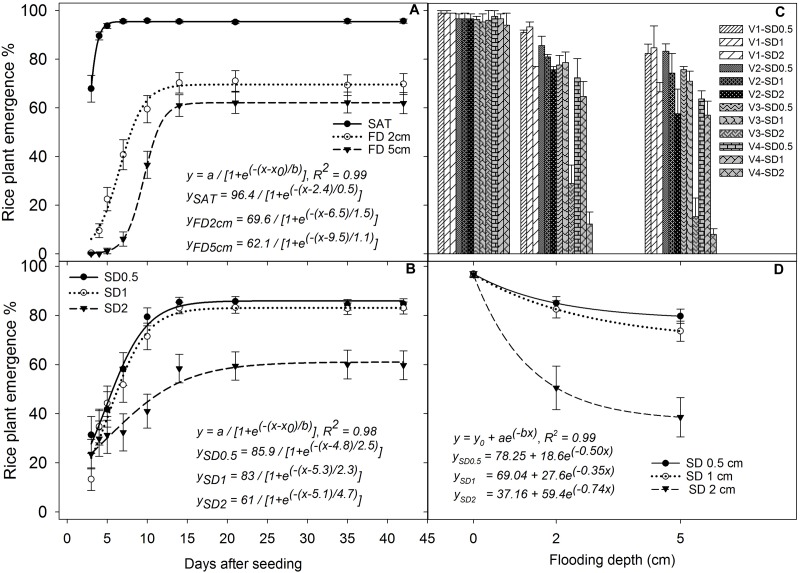
Percentage seedling emergence **(A)** under different flooding depths irrespective of sowing depth and genotype, **(B)** at different sowing depths irrespective of flooding depth and genotype, **(C)** of four genotypes under different sowing and flooding depths at 35 DAS, and **(D)** under different sowing and flooding depths at 35 DAS irrespective of genotype. The lines in **(A,B)** represent three-parameter sigmoid model *y* = *a*/{1 + *e*^(−(^*^x-x0^*^)/^*^b^*^)^} fitted to percentage rice emergence; and the lines in **(D)** represent a three-parameter exponential decay model [*y* = *y*_0_ + *ae*^(^*^−bx^*^)^] fitted to percentage rice emergence. Vertical bars represent standard error of the mean. V1, Khao Hlan On; V2, Ma-Zhan Red; V3, IR64+AG1; V4, IR64; SAT, saturated; FD2cm, 2 cm flooding depth; FD5cm, 5 cm flooding depth; SD0.5, 0.5 cm sowing depth; SD1, 1 cm sowing depth; SD2, 2 cm sowing depth.

The main and interaction effects of FD, SD, and genotype on seedling emergence above water were all significant (*P* < 0.05; Figure 2). Under saturated conditions, the emergence of rice seedlings above the soil surface was first observed at 2 DAS and reached the maximum by 5 DAS (Figure 2A), and all the genotypes had more than 96% emergence (*P* > 0.05) irrespective of SD and genotype. The initial emergence of rice seedlings above the soil surface was faster when SD was shallower (SD 0.5 cm and 1 cm) (Figure 2B). A delay in rice emergence above the water surface was observed under 2 cm and 5 cm FDs compared with saturated conditions, and percentage emergence reached its maximum by 14–18 DAS (Figure 2A). There were no further improvements in percentage emergence of rice seedlings even after draining water at 28 DAS. The maximum rice seedling emergence estimated from the fitted model under 2 cm FD was 69.6%, which was 28% lower than that under saturated conditions and this reduction reached 37% with a further increase in FD to 5 cm (Figure 2A). There was no significant difference (*P* > 0.05) in emergence between 0.5 and 1 cm SD at 21 DAS (Figure 2B). However, percentage emergence decreased significantly when the SD increased to 2 cm under 2 cm and 5 cm FD (Figures 2B,D). At 35 DAS, the fitted model showed a reduction in emergence of rice seeds sown under 2 cm SD by 41% and 51%, respectively, at 2 cm and 5 cm FD compared with that of seeds sown under 0.5 cm SD at similar FD (Figure 2D). This reduction was 48% and 60%, respectively, when compared with that under saturated conditions. In addition, rice plant emergence decreased by 12–15% and 14–22% when the seeds were sown at 0.5 cm and 1 cm SD under 2 cm and 5 cm FD, respectively, compared with that under saturated conditions (Figure 2D).

#### Responses of Genotypes to Sowing and Flooding Depths

The four rice genotypes showed similar performance in percentage emergence under saturated conditions at 35 DAS, but significant variation (*P* < 0.05) was observed under flooded treatments (Figures 2C,D). At 35 DAS, rice emergence within the same genotype did not vary (*P*> 0.05) with flooding treatments when plants were sown at 0.5 cm and 1 cm SD except for IR64, in which % emergence decreased by 30% and 22–40% with an increase in FD from saturated to 2 cm and 5 cm, at SD of 0.5 cm and 1.0 cm, respectively. Percentage emergence decreased significantly in all genotypes when the SD was increased from 1 to 2 cm. Khao Hlan On showed the highest percentage emergence under all flooding and SD combinations. Under 2 cm flooding, the percentage emergence of Khao Hlan On decreased by 8%, 6%, and 23% at 0.5 cm, 1 cm, and 2 cm SD, respectively, and by 13%, 15%, and 33%, respectively, under 5 cm FD compared with that of saturated conditions (Figure 2C). The AG-tolerant genotypes Khao Hlan On and Ma-Zhan Red showed similar (*P* > 0.05) percentage emergence when the seeds were sown at 0.5 cm SD under flooded conditions, whereas IR64 showed a 30% and 25% reduction in emergence (*P* < 0.05) under the same conditions compared with Khao Hlan On and Ma-Zhan Red at 35 DAS (Figure 2C). IR64+AG1 did not vary significantly (*P*> 0.05) from the other genotypes as it showed intermediate responses between tolerant and moderately tolerant genotypes under similar conditions. At 2 cm FD, when the seeds were sown at 1 cm depth, Khao Hlan On had the highest percentage emergence. However, a 24% reduction in emergence was shown by IR64 compared with Khao Hlan On, whereas Ma-Zhan Red and IR64+AG1 were similar (*P* > 0.05) and ranked between Khao Hlan On and IR64. At 35 DAS, all three tolerant genotypes (i.e., Khao Hlan On, Ma-Zhan Red, and IR64+AG1) performed similarly when sown at 1 cm SD under 5 cm FD, whereas emergence of IR64 decreased by 33% compared with the tolerant genotypes.

The emergence of all rice genotypes declined significantly (*P*< 0.05) when SD was increased to 2 cm, with Khao Hlan On and Ma-Zhan Red performing similarly, resulting in 77% emergence, whereas the emergence of IR64+AG1 and IR64 was 32% and 8%, respectively, at 2 cm FD (Figure 2C). Moreover, the seedling emergence of IR64+AG1 and IR64 was 66% and 94% lower, respectively, under 2 cm FD when sown to a depth of 2 cm than that under saturated conditions. Under the more severe stress conditions (2 cm SD and 5 cm FD), the tolerant genotypes Khao Hlan On and Ma-Zhan Red showed similar percentage emergence (67% and 61%, respectively; *P*> 0.05), whereas emergence of both IR64+AG1 and IR64 declined drastically, to less than 15%.

Genotypes Khao Hlan On and Ma-Zhan Red have thick pericarp as a clear distinguishable morphological trait, which does not exist in the other two genotypes. The pericarp can protect the kernel against external agents, including insects or microorganisms, and help conduct and distribute water and other nutrients during the germination process (Serna-Saldivar, 2010). In addition, most of the protective phenolic compounds are also associated with the pericarp cells, which help prolong viability of the seeds under submerged conditions (Serna-Saldivar, 2010; Mora et al., 2013). Therefore, in addition to the inherent traits of flood-tolerance mechanisms (Angaji et al., 2010), the thick pericarp could be related to Khao Hlan On and Ma-Zhan Red having higher percentage emergence under all SD and FD than IR64+AG1 and IR64, which do not contain a thick pericarp. Therefore, the use of appropriate seed coating agents that help prolong the seed viability under flooded conditions is suggested as these might act similar to the seed pericarp in enhancing percentage emergence by reinforcing the tolerance of AG of genotypes.

The results revealed that rice seedling emergence was more adversely affected by flooding when seeds were sown at 2 cm SD than when the SD was 1 cm or less (Figures 2C,D). The redox potential near the soil surface of a flooded soil is around +400 mv, which decreases to −300 mv at around 1 cm soil depth in flooded soils even though the oxygen concentration does not reach zero within this range (Pezeshki and De Laune, 2012). Therefore, the first 1 cm soil layer is mostly hypoxic, with low but available oxygen for oxidization reactions. However, the redox potential beyond 1 cm soil depth declines drastically with no available oxygen (highly anaerobic or anoxic), as all the reduction reactions occur in this layer (Ponnamperuma, 1975a,b, 1981; Pezeshki and De Laune, 2012). The other consequences of soil reduction processes include changes in availability and concentrations of various nutrients that are essential for plant functions and the production of a host of compounds phytotoxic for plant growth (Ponnamperuma, 1975a,b; Tsutsuki and Ponnamperuma, 1987; Armstrong and Drew, 2002; Pezeshki and De Laune, 2012). Therefore, it is likely that the high level of oxygen depletion at SD deeper than 1 cm from the soil surface and the accumulation of toxic compounds due to unfavorable soil chemical reactions under reduced conditions could be the main reasons for the poor crop establishment of all genotypes when seeds were sown at 2 cm depth under flooded conditions.

Furthermore, we observed a weak vertical stand of the plants despite good emergence when sown at shallow SD of 0.5 cm under flooded conditions. This happened because of the higher possibility of exposure of the upper region of the germinating seed, or the region that connects the roots with the stem base of developing seedlings, above the soil surface when the SD was shallow (0.5 cm) under flooded conditions. Hence, the rice plant stand was weaker and was vulnerable to lodging with slower external wind forces. In addition, floating of young seedlings above the water surface was also a concern when the seeds were sown at 0.5 cm depth under flooded conditions. This could be because, when rice seed germinates under such a low oxygen environment (flooded conditions), coleoptile elongation is promoted while radical elongation and root development are inhibited until the tip of the coleoptile reaches a high oxygen concentration region (Vartapetian and Jackson, 1997; Huang et al., 2003; Magneschi and Perata, 2009; Miro and Ismail, 2013). The developing coleoptile possesses more porous inner tissues called aerenchyma and the buoyant force generated by the porous tissues is greater than normal diffusion forces (Vartapetian and Jackson, 1997). The high buoyant force, lack of roots to anchor seedlings strongly to the soil during early seedling growth, and lower downward force on the seed by the thin layer of soil would have resulted in exposure of seeds of developing seedlings or floating of young seedlings above the water surface when the seeds were sown at 0.5 cm depth under flooded conditions. Therefore, 1 cm SD is recommended to obtain better crop stand/establishment under flooded conditions for AG-tolerant genotypes to facilitate better crop growth.

### Plant Height

**FIGURE 3:**
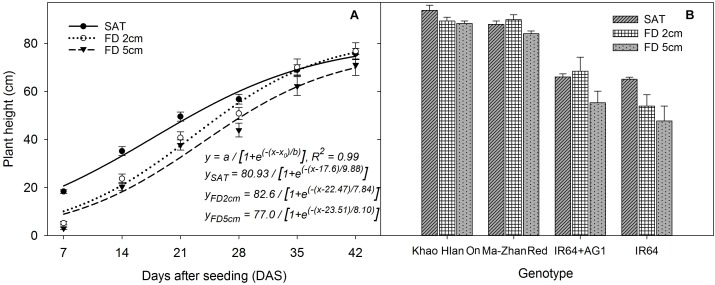
**(A)** Plant height of rice (average of four genotypes) from 7 to 42 DAS under different flooding depths irrespective of sowing depth and **(B)** plant height of different genotypes under different flooding depths irrespective of sowing depth at 42 DAS. Lines in **(A)** represent three-parameter sigmoid model *y* = *a*/{1 + *e*^(−(*x-x*0)/*b*)^} fitted to plant height. The vertical bars represent standard error of the mean. SAT, saturated; FD2cm, 2 cm flooding depth; FD5cm, 5 cm flooding depth.

The main and interaction effects of FD, SD, and rice genotype on plant height from 7 to 21 DAS were significant (*P* < 0.05) (Supplementary Table S1). However, the plant height from 28 to 35 DAS was affected by main effects of FD, SD, and genotype and the interaction effects of FD × SD, FD × genotype, and SD × genotype. At 42 DAS, plant height was significantly influenced by SD, genotype, and their interaction (SD × genotype). The sigmoid model fitted to plant height resulted in an *R*^2^ = 0.99 (*P* < 0.05). The simulated values derived through the regression analysis showed that the final plant height (parameter *a*) of rice reached 80 cm, 82 cm, and 77 cm at saturated, 2 cm, and 5 cm FD, respectively, irrespective of the SD used (Figure 3A). The time to reach 50% of final plant height (parameter *x*_0_) was 18, 23, and 23.5 days at saturated, 2 cm, and 5 cm FD, respectively. The rate of plant height increase (slope of the curve; parameter *b*) was significant (*P* < 0.05) under all flooding conditions imposed and was 9.88%, 7.84%, and 8.10% under saturated, 2 cm, and 5 cm FD, respectively. Among the four rice genotypes evaluated in this study, Khao Hlan On and Ma-Zhan Red were similar in plant height under different FD at 35 DAS and grew taller than IR64+AG1 and IR64, which also showed a similar plant height under saturated and 2 cm FD, but their height varied under 5 cm FD depending on the tolerance level (Figure 3B).

Carbohydrate metabolism and cell division are inhibited or slowed down in germinating rice seeds under submerged conditions because of the low activity of the enzymes involved in these processes under anaerobic environments (Miro and Ismail, 2013). Under submerged conditions, the starch concentration of germinated rice seeds either does not vary significantly in the sensitive genotypes or progressively decreases with time in the tolerant genotypes at a comparatively slower rate than in those grown under aerobic conditions (Ismail et al., 2009), resulting in late emergence and suppressed initial seedling growth. These could be some of the reasons for lower initial plant height and the slower rate of shoot elongation under flooded conditions than under saturated conditions. However, at 14–18 DAS, plant height showed an increasing trend, which was observed soon after the seedlings emerged above the water surface (Figure 3A). Soluble sugars are available at slow but increasing concentrations even as late as 8 DAS when the seeds germinate under flooded conditions (Ismail et al., 2009), and that could subsequently be used in glycolysis to produce more ATP (Miro and Ismail, 2013), together with the activation of many enzymes and growth-promoting hormones soon after seedlings emerge above the water surface. This could be the main reason that faster growth was triggered under flooded conditions at and beyond 14 DAS in this study. These results and those of the fitted regression model clearly indicate that the height of rice plants grown under 2 cm FD reached the height of those grown under hypoxic conditions (saturated conditions) at 42 DAS. Similarly, the plants grown under 5 cm FD also showed faster growth at 14–18 DAS than those grown under saturated conditions.

### Leaf Area Production

**FIGURE 4:**
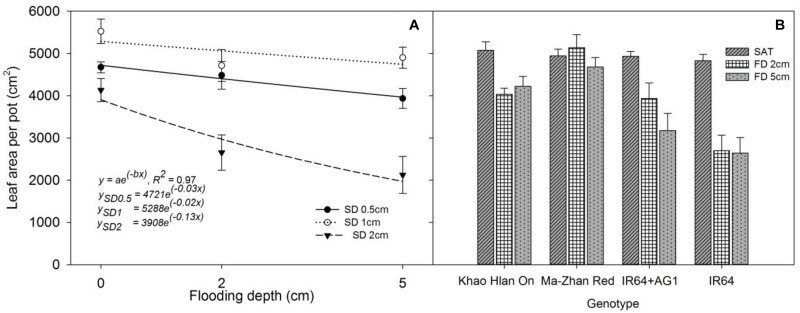
Leaf area of **(A)** rice genotypes at different sowing and flooding depths and **(B)** of different genotypes at different flooding depths. Data are taken at 42 DAS. The lines in **(A)** represent a two-parameter exponential decay model [*y* = *ae*^(^*^−bx^*^)^] fitted to leaf area measurements of rice. The vertical bars represent standard error of the mean. SD0.5, 0.5 cm sowing depth; SD1, 1 cm sowing depth; SD2, 2 cm sowing depth.

Leaf area production per pot at 42 DAS was significantly influenced (*P*< 0.05) by treatments and the interaction effect of FD × SD × genotype (Figures 4A,B). All genotypes produced similar leaf area (*P*> 0.05) under saturated conditions. At 2 cm and 5 cm FD, the highest leaf area was observed in Ma-Zhan Red (*P*< 0.05) under all SD, whereas Khao Hlan On and IR64+AG1 showed similar results (*P*> 0.05) under both 2 cm and 5 cm FD when sown at 1 cm SD (Figure 4B). Under all flooding treatments, all genotypes produced maximum leaf area when sown at 1 cm SD compared with 0.5 cm and 2 cm SD (*P*< 0.05). The reduction in leaf area of Khao Hlan On, IR64+AG1, and IR64 was 20.4%, 20.1%, and 44.1%, respectively, under 2 cm FD compared with that of saturated conditions, irrespective of the SD (Figure 4B). Compared to saturated conditions, the leaf area of Ma-Zhan Red and Khao Hlan On was 4% and 16% lower, respectively, at 5 cm FD, whereas the leaf area of IR64+AG1 and IR64 decreased by 35% and 45%, respectively (Figure 4B). The leaf area of IR64+AG1 and IR64 was significantly lower than that of Khao Hlan On and Ma-Zhan Red under FD of 5 cm and SD of 2 cm (*P*< 0.05). The rice genotypes IR64+AG1 and IR64 showed similar performances under 5 cm FD when the plants were grown at 0.5 cm and 1 cm SD, but showed a 47% and 66% reduction (*P*< 0.05) in leaf area production, respectively, when grown at 2 cm SD compared with plants grown at 1 cm SD. At 2 cm FD, Khao Hlan On and Ma-Zhan Red showed 16% and 13% higher leaf area production, respectively, when sown at 1 cm depth than at 0.5 cm, and 22% and 27% greater leaf area, respectively, than when grown at SD of 2 cm.

The exponential decay model fitted to leaf area production resulted in an *R*^2^ = 0.97 (*P*< 0.05). At 42 DAS, leaf area was highest when SD was 1 cm, followed by when SD was 0.5 cm, and was least when plants were sown at 2 cm SD at all FDs (Figure 4A). The regression analysis indicated that the maximum leaf area production (parameter *a*) reached 4,721 cm^2^, 5,288 cm^2^, and 3,908 cm^2^ at 0.5 cm, 1 cm, and 2 cm SD, respectively, under saturated conditions. Moreover, a higher rate of decrease (parameter *b*) in leaf area production was recorded when the seeds were sown at 2 cm (*b* = −0.13) depth than for those at 0.5 cm (*b* = −0.03) and 1 cm (*b* = −0.02) SD with an increase in FD.

Khao Hlan On and Ma-Zhan Red are landraces with droopy and comparatively long leaves (Septiningsih et al., 2013a) compared with modern rice genotypes. In addition, both genotypes are highly tolerant of flooding during germination, resulting in a comparatively higher percentage of seedling emergence (more plants per pot) under flooded conditions. They were also reported to have fast coleoptile elongation, facilitating fast initial seedling growth (Septiningsih et al., 2009). All of these characters may have contributed to the comparatively higher leaf area of Khao Hlan On and Ma-Zhan Red than that of IR64+AG1 and IR64, especially under higher flooded conditions. IR64+AG1 also showed similar capacity of leaf area production under 2 cm FD when sown at SD of 0.5 cm and 1 cm, although the number of plants per pot was lower than that of Khao Hlan On and Ma-Zhan Red, thus highlighting its relative competitive ability. The declining leaf area production of IR64+AG1 with the increase in FD to 5 cm could be due to the lower emergence percentage (fewer plants per pot) and slow growth under deeper floods. Higher tolerance than that conferred by AG1 alone is probably required to withstand deeper floods during crop establishment. The 2 cm SD had a greater effect on emergence and plant growth, including leaf area production, as clearly demonstrated by lowering the performances of IR64+AG1 and IR64 when the plants were grown at 2 cm SD under higher FD.

### Tiller Density per Pot

ANOVA indicated that tiller density per pot was significantly influenced (*P*< 0.05) by the interaction effect of FD × SD × rice genotype at 42 DAS. There was no significant difference in tiller density between Khao Hlan On, Ma-Zhan Red, and IR64+AG1 at 0.5 and 1 cm SD under saturated conditions and FD of 2 cm and 5 cm. However, at 2 cm FD, Khao Hlan On and Ma-Zhan Red did not show a significantly different tiller density at all SD whereas IR64+AG1 and IR64 showed a decrease in tiller density by 58% and 88%, respectively, at 2 cm SD under 2 cm FD, compared with plants grown at 1 cm SD. Khao Hlan On, Ma-Zhan Red, IR64+AG1, and IR64 showed a decline in tiller density (*P*< 0.05) by 30%, 28%, 72%, and 78%, respectively, when grown at 2 cm SD under 5 cm FD compared with plants grown under saturated conditions (data not presented).

**FIGURE 5:**
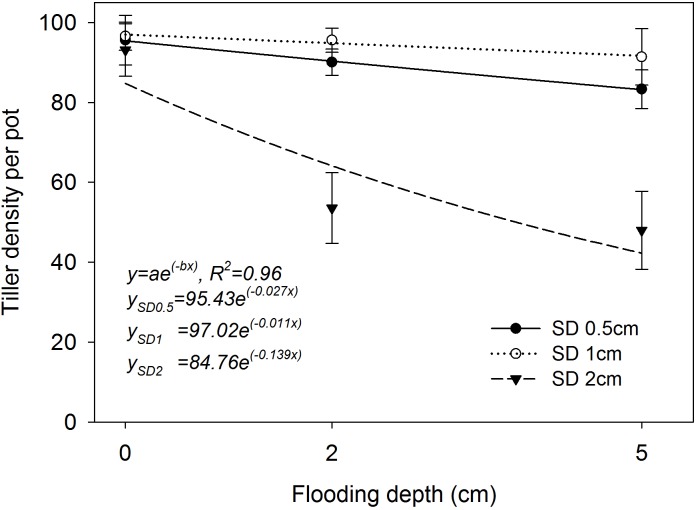
Tiller density per pot at different sowing depths and under different flooding depths. Data are averages across genotypes collected at 42 DAS. The lines represent a two-parameter exponential decay model [*y* = *ae*^(^*^−bx^*^)^] fitted to tiller density. The vertical bars represent standard error of the mean. SD0.5, 0.5 cm sowing depth; SD1, 1 cm sowing depth; SD2, 2 cm sowing depth.

The two-parameter exponential decay model fitted to the tiller production of rice plants at 42 DAS resulted in *R*^2^ = 0.96 (*P*< 0.05; Figure 5). The simulated values derived from the fitted model highlighted the highest tiller density of 97 per pot (parameter *a*) when the plants were grown at 1 cm SD under saturated conditions. The plants that were grown at 0.5 cm and 2 cm SD yielded 95 and 84 tillers per pot, respectively. A drastic reduction in tiller production (−0.139; parameter *b*) was observed when the SD was increased to 2 cm under flooding. The lowest rate of reduction in tiller density with an increase in FD was observed when the plants were grown at 1 cm SD (*b* = −0.011), followed by 0.5 cm SD (*b* = −0.027) and 2 cm (*b* = −0.139) SD. All genotypes had higher tiller density when sown at 1 cm SD under flooded and saturated conditions (Figure 5).

The fitted model highlighted the lowest as well as highest rate of reduction in tiller production of AG genotypes when seeds were sown at 2 cm under higher FD of 5 cm. Compensatory growth is an inherent trait of rice under environmental stresses (Kyu et al., 2002) and also for pest damage (Muralidharan and Pasalu, 2006) at the vegetative stage, by producing new tillers and leaves. Even though percentage emergence decreased by 14–22% at 1 cm SD under 2 cm and 5 cm FD (Figure 2D), the emerged rice plants were able to produce a similar number of tillers per pot compared with those under saturated conditions in this study, highlighting the compensatory growth habit of rice. Therefore, with this compensatory growth, it could be predicted that rice plants grown at 1 cm SD under flooded conditions will produce a similar yield to that of plants grown under saturated conditions. Rice plants are susceptible to lodging when the base of the plant is weakly anchored to the soil due to shallow seeding and this would result in reduced crop growth and yield (Vergara, 1992). Similarly, the low tiller production in plants grown at 0.5 cm SD under flooded conditions in this study could be due to weak stand.

### Total Shoot Biomass per Pot

**FIGURE 6:**
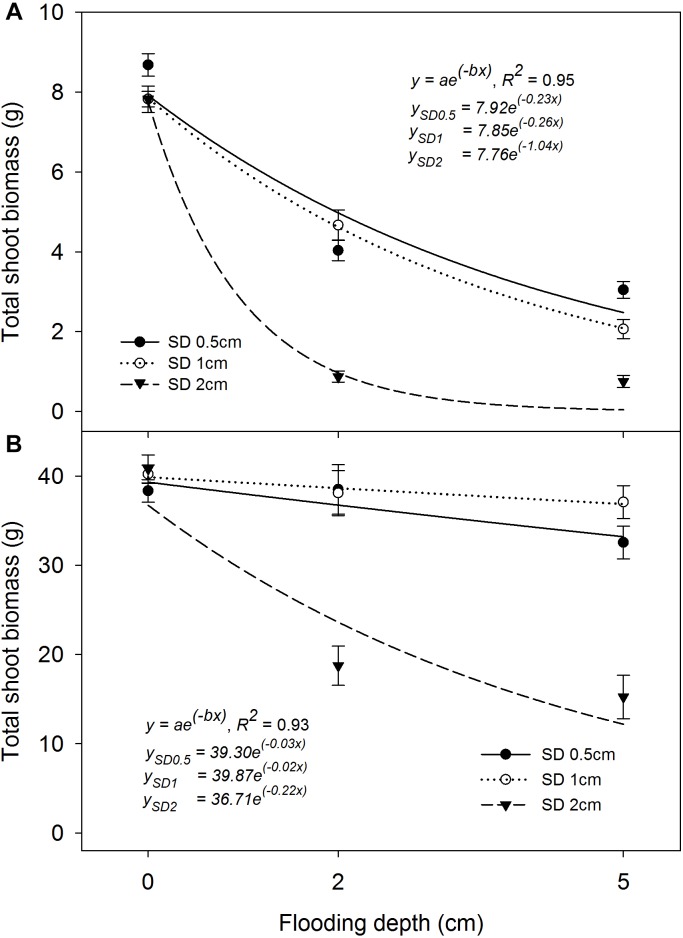
Total shoot biomass (TSBM) per pot at **(A)** 21 DAS and **(B)** 42 DAS under different flooding and sowing depths irrespective of genotype. The lines in **(A,B)** represent a two-parameter exponential decay model [*y* = *ae*^(^−bx^)^] fitted to rice TSBM. The vertical bars represent standard error of the mean. SD0.5, 0.5 cm sowing depth; SD1, 1 cm sowing depth; SD2, 2 cm sowing depth.

Total shoot biomass per pot at 21 and 42 DAS was significantly influenced by the interaction effect of FD × SD × rice genotype (*P*< 0.05). The exponential decay model fitted to TSBM production at 21 and 42 DAS showed *R*^2^ = 0.95 and *R*^2^ = 0.93, respectively (*P*< 0.05; Figure 6). At 21 DAS, TSBM production at 0.5 cm and 1 cm SD was lower by 51–63% and 43–74%, respectively, under 2 cm and 5 cm FD, compared with that of saturated conditions. An 88% and 90% lower TSBM production was observed when plants were sown at 2 cm under 2 cm and 5 cm FD, respectively, compared with that under saturated conditions. The fitted model showed a similar rate of decrease (parameter *b*) in TSBM production when the plants were grown at 0.5 cm (*b* = −0.23) and 1 cm (*b* = −0.26) SD. However, a comparatively greater rate of decrease at 2 cm SD (*b* = −1.04) with increasing FD at 21 DAS was observed. At 42 DAS, TSBM production under flooded conditions showed an increasing trend compared with that of saturated conditions at 21 DAS (Figures 6A,B). The increase in TSBM during the period between 21 and 42 DAS was 1.51 g pot^−1^ day^−1^ under saturated conditions and was 1.62 g pot^−1^ day^−1^ and 1.53 g pot^−1^ day^−1^ for plants grown at 0.5 cm or 1 cm SD under 2 cm and 5 cm FD, respectively. The average rate of increase in TSBM when the seeds were sown at 2 cm was 0.85 g pot^−1^ day^−1^ and 0.69 g pot^−1^ day^−1^ at 2 cm and 5 cm FD, respectively, which was 48% and 55% lower than for seeds sown at 1 cm SD under 2 cm and 5 cm FD, respectively. The simulated values of the fitted model showed maximum TSBM production of 39.9 g pot^−1^ (parameter *a*) when the plants were grown at 1 cm SD at 42 DAS under saturated conditions. At 42 DAS, the rate of decrease in TSBM of the fitted model (parameter *b*) with an increase in FD was lower for rice grown at 1 cm SD (*b* = −0.02) than for rice grown at 0.5 cm SD (*b* = −0.03). The rate of decrease in TSBM production was far greater (*b* = −0.22) when SD was increased to 2 cm under different FDs. In contrast, TSBM production of plants grown at 0.5 cm SD was higher at 21 DAS (Figure 6A) than for plants grown at 1 cm SD; however, the highest TSBM production at 42 DAS was reported from plants grown at 1 cm SD (Figure 6B).

At 42 DAS, all genotypes showed similar TSBM production (*P*> 0.05) when the plants were grown at 1 cm SD under saturated conditions (∼40 g pot^−1^). Khao Hlan On and Ma-Zhan Red did not differ in TSBM under each FD of 2 cm and 5 cm, irrespective of the SD (*P*> 0.05). At 2 cm FD, both IR64+AG1 and IR64 showed similar (*P*> 0.05) TSBM at each SD of 1 cm (∼27 g pot^−^1) and 2 cm (∼10 g pot^−1^), which is, however, lower by 32% and 75%, respectively, than for plants grown under saturated conditions. At 5 cm FD, IR64+AG1 TSBM production was similar to that of Khao Hlan On and Ma-Zhan Red when grown at 1 cm SD. In contrast, all genotypes showed significantly lower TSBM production under flooded conditions when sown at 2 cm than at 0.5 cm and 1 cm (Figure 6). At 2 cm FD, TSBM of Khao Hlan On, Ma-Zhan Red, IR64+AG1, and IR64 grown under 2 cm SD were lower by 37%, 48%, 63%, and 65%, respectively, whereas, at 5 cm FD, the reductions were even higher (at 41%, 52%, 84%, and 90%, respectively) than those at 1 cm SD. The change in the trend of higher TSBM production at 42 DAS than at 21 DAS could be related to better anchorage when plants were sown at 1 cm under flooded conditions, resulting in better crop growth than for plants sown at 0.5 cm (Reeve, 2013). The 2 cm SD is non-conducive for rice plant emergence, growth, and biomass production under flooded conditions as evident in this study, which could be due to the anoxic conditions under deeper layers of submerged soils (Armstrong and Drew, 2002), and poor seedling vigor and slow initial vegetative growth of genotypes under flooded conditions. The comparatively higher rate of biomass accumulation for plants grown under flooded conditions than under saturated conditions highlights the compensatory growth potential of rice in overcoming such stress conditions (Kyu et al., 2002).

### Leaf Number per Plant

**FIGURE 7:**
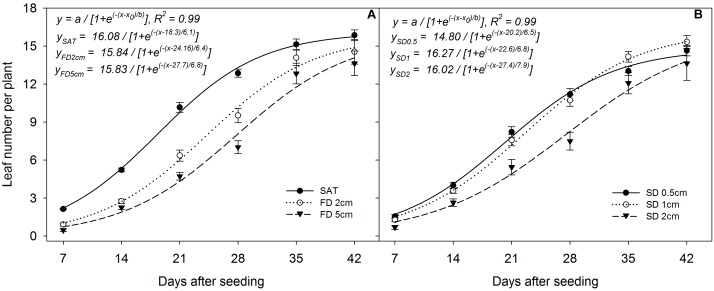
Leaf number per plant when grown **(A)** at different flooding depths and **(B)** under different sowing depths averaged across genotypes. The lines represent three-parameter sigmoid model *y* = *a*/{1 + *e*^(−(*x-x*0)/*b*)^} fitted to rice plant height. The vertical bars represent standard error of the mean. SAT, saturated; FD2cm, 2 cm flooding depth; FD5cm, 5 cm flooding depth; SD0.5, 0.5 cm sowing depth; SD1, 1 cm sowing depth; SD2, 2 cm sowing depth.

The sigmoid regression model fitted to leaf number per plant at different FD and SD resulted in an *R*^2^ = 0.99 in both situations (*P*< 0.05; Figures 7A,B). The simulated maximum leaf number per plant (parameter *a*) was 16.08, 15.84, and 15.83 (Figure 7A) and the time to reach 50% of the final leaf number per plant was 18.3, 24.16, and 27.7 days at saturated, 2 cm, and 5 cm FD, irrespective of SD and genotype. However, the rate of increase in leaf number per plant was higher at 5 cm FD (*b* = 6.8) than at 2 cm FD (*b* = 6.4) and in saturated conditions (*b* = 6.1). The regression model showed that sowing seeds at 1 cm depth (parameter *a*) had the maximum leaf number per plant (*a* = 16.27), followed by 2 cm (*a* = 16.02), with the lowest at 0.5 cm (*a* = 14.80), irrespective of the genotype (Figure 7B). The time to reach 50% of maximum leaf number per plant was 20.2, 22.6, and 27.4 days at 0.5 cm, 1 cm, and 2 cm SD, respectively. The rate of increase in leaf number per plant was higher for plants sown at 2 cm (*b* = 7.9) than at 1 cm (*b* = 6.8) and 0.5 cm (*b* = 6.5).

Apparently, early plant growth under flooded conditions is very slow, especially during the first 14 days (Figure 7A). However, the final leaf number per plant (parameter *a*) is a clear indication that plants sown under flooded conditions would compensate and reach almost similar leaf number per plant as under saturated conditions. The initial lag period of 14 days and greater anaerobic stress under 5 cm water layer could be the reason for the comparatively longer duration to reach 50% of the final maximum leaf number per plant than for plants under shallower flooding.

The weak vertical stand due to insufficient anchorage to the soil might be the reason for the comparatively lower leaf number per plant for plants sown at 0.5 cm under flooded conditions. Rice plants sown at 0.5 cm have also shown comparatively higher leaf production at the early phase (until 28 DAS) of growth, which started declining at later stages, highlighting the impact of poor anchorage. Growth was affected when plants were sown at 2 cm, especially under flooded conditions, mainly because of greater anaerobic stress. The fitted model showed a higher rate of increase in leaf number per plant for plants sown at 2 cm, which could be due to the compensatory response of attempting to overcome retarded growth brought about by anaerobic conditions during flooding (Kyu et al., 2002).

### Weed Emergence Percentage

**FIGURE 8:**
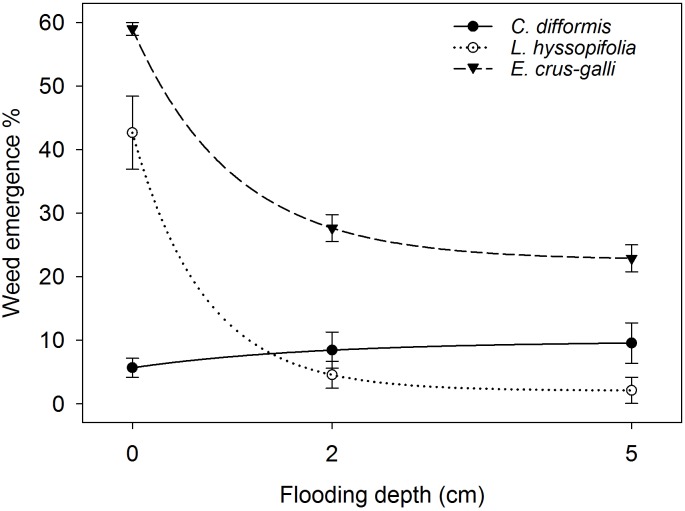
Weed emergence percentage under different flooding depths at 21 DAS. The lines represent a three-parameter exponential decay model [*y* = *y_0_ + ae*^(^*^−bx^*^)^] fitted to weed emergence %. The vertical bars represent standard error of the mean.

ANOVA indicated that percentage weed emergence was significantly influenced by FD (*P*< 0.05) (Figure 8). The regression model fitted to % weed emergence resulted in an *R*^2^ = 0.96 (*P*< 0.05). The maximum % weed emergence (parameter *a*) under saturated conditions was 42.39, 59.70, and 7.50 for *L. hyssopifolia*, *E. crus-galli*, and *C. difformis* under saturated conditions. However, in contrast to the grass (*E. crus-galli*) and broadleaf (*L. hyssopifolia*), the sedge (*C. difformis*) weed species showed 49% and 68% higher emergence under flooded conditions than under saturated conditions. The 2 cm and 5 cm FD suppressed the emergence of *L. hyssopifolia* by 89% and 95%, respectively, and of *E. crus-galli* by 53% and 61%, respectively, compared with saturated conditions. However, the emergence of all of the tested weed species did not differ at 2 cm and 5 cm FD (*P*> 0.05).

Weed seed distribution was uniform within the topmost 0.5 cm soil layer. Therefore, there was equal chance to establish the same percentage of seeds on the surface as well as in different depths within 0.5 cm from the soil surface, which was similar to field conditions. Previous studies have shown that emergence of *E. crus-galli* decreased by 50% when the burial depth increased to 0.4 cm (Chauhan and Johnson, 2011), whereas emergence of *L. hyssopifolia* and *C. difformis* declined by 80% and 99%, respectively, when the burial depth increased to 0.5 cm compared with that of surface-sown weed seeds (Chauhan and Johnson, 2009a,b). Hence, variation in burial depth between the surface and 0.5 cm depth might be the reason for the very low emergence of *C. difformis*, about 40% less seedling establishment than *E. crus-galli* and 67% less seedling establishment than *L. hyssopifolia* under saturated conditions compared with that of weed seed germination inside the incubator as observed in this study. The 53% reduction in the seedling establishment of *E. crus-galli* at 2 cm FD as observed in this study is in contrast to the findings of Estioko et al. (2014), who reported that 2 cm of flooding did not reduce the seedling establishment of this weed significantly compared with that of non-flooded conditions. The comparable soil profile (8.8 kg soil per pot with 20 cm depth) with that of field conditions, which simulated the real situation of soil chemical reactions and higher anaerobic stress (Armstrong and Drew, 2002; Pezeshki and De Laune, 2012), and the weed seed distribution within the 0.5 cm soil layer may be the reasons for the reduced seedling establishment of *E. crus-galli* in this study. *Cyperus difformis* is an obligate aquatic weed species and therefore is difficult to control through water management (Caton et al., 2002; Yaduraju and Mishra, 2008). It showed an increased percentage emergence under flooded conditions in this study. Moreover, this sedge does not emerge well under aerobic conditions (Yaduraju and Mishra, 2008). Hence, the overall poor emergence of *C. difformis* could be because of its sensitiveness to seed burial depth (Chauhan and Johnson, 2009a), and its comparatively lower emergence under saturated conditions could be attributed to its obligate aquatic nature. *Echinochloa crus-galli* has some flood tolerance and demonstrated the ability to detoxify acetaldehyde generated during anaerobic fermentation through an enhanced level of aldehyde dehydrogenase activity (Estioko et al., 2014). In addition, the comparatively higher percentage of the emergence of *E. crus-galli* compared with that of *L. hyssopifolia* and *C. difformis* under flooded conditions could be related to its relatively larger seed size (Colmer et al., 2014), as seeds may have sufficient food reserves to support seedling emergence.

### Weed Biomass Production

**FIGURE 9:**
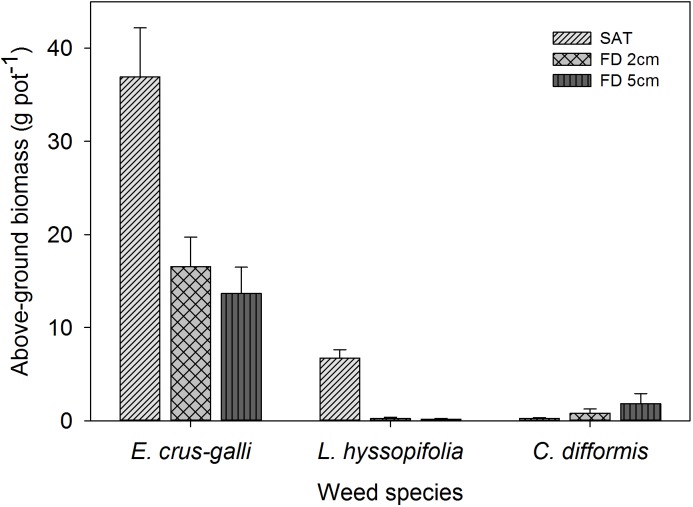
Aboveground weed biomass of *E. crus-galli*, *L. hyssopifolia*, and *C. difformis* at 42 DAS. The vertical bars represent standard error of the mean. SAT, saturated; FD2cm, 2 cm flooding depth; FD5cm, 5 cm flooding depth.

The AGB production of all weed species was significantly influenced by FD (*P*< 0.05) at 42 DAS (Figure 9). *Cyperus difformis* produced higher biomass with increased FD to 2 cm and 5 cm (0.8 g pot^−1^ and 1.8 g pot^−1^, respectively). In addition, the AGB production of *C. difformis* under saturated conditions (0.24 g pot^−1^) was lower by 70% and 85% than that of the 2 cm and 5 cm FD, respectively. *Ludwigia hyssopifolia* produced significantly higher biomass under saturated conditions (6.74 g pot^−1^), which then decreased by 96% at 2 cm FD and by 98% at 5 cm FD (*P*< 0.05).

**FIGURE 10:**
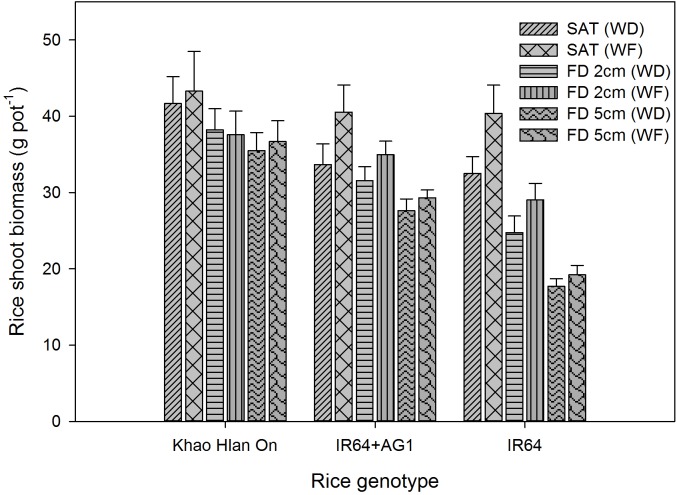
Genotypic variation in shoot biomass as affected by flooding and weed competition at 42 DAS. The vertical bars represent standard error of the mean. SAT, saturated; FD2cm, 2 cm flooding depth; FD5cm, 5 cm flooding depth; WD, weedy; WF, weed-free.

Khao Hlan On decreased the AGB of *L. hyssopifolia* by 43% under saturated conditions compared with when the weed was in association with IR64+AG1 and IR64 (data not shown). However, all three rice genotypes had a similar effect (*P*> 0.05) in reducing the shoot biomass of *L. hyssopifolia* when grown under 2 cm and 5 cm FD. The AGB of *E. crus-galli* was significantly higher under saturated conditions (36.9 g pot^−1^), which was closer to that of the shoot biomass of rice (Figure 10) at 42 DAS. However, the shoot biomass of *E. crus-galli* decreased by 55% and 62% under 2 cm and 5 cm FD, respectively (Figure 9). The tolerant genotype Khao Hlan On showed the greatest weed-competitive ability under saturated and flooded conditions. The shoot biomass of *E. crus-galli* declined by 59% and 48% under saturated conditions and 5 cm FD, respectively, when grown with Khao Hlan On, compared (*P*< 0.05) with that of *E. crus-galli* grown in association with IR64+AG1 and IR64. IR64+AG1 and IR64 showed similar abilities in reducing the biomass of *E. crus-galli* under flooded conditions.

The AGB production of *C. difformis* is in line with the findings of Chauhan and Johnson (2009a) and further highlights the requirement for deeper/higher flooding to have better control of the weed. The reduction in the AGB of *L. hyssopifolia* by flooding observed in this study has also been reported by Chauhan and Johnson (2009b). Grassy weeds constitute about 80% of the total weed community in DSR (Anwar et al., 2012) and the establishment of *E. crus-galli* and other common grass weeds are greater under saturated conditions (Bhagat et al., 1999). Similarly, a higher percentage of emergence and higher AGB were observed under saturated conditions in this study. Khao Hlan On is a landrace rice genotype (Kretzschmar et al., 2015), which has faster seedling growth and higher plant height than IR64+AG1 and IR64, categorized as semi-dwarf types (Kerdphol et al., 2015). Therefore, the inherent characteristics of Khao Hlan On have contributed to smothering weeds with good ground cover under saturated and flooded conditions (5 cm FD). Hence, the results highlighted the importance of improving the AG tolerance of IR64+AG1 for better performance under deeper/higher flooding to suppress weed emergence and growth without hampering rice establishment. Sunyob et al. (2015) showed that weed biomass production for semi-dwarf rice genotypes is greater in DSR. Moreover, semi-dwarf rice genotypes exhibit poor seedling vigor, which is associated with insufficient mesocotyl elongation at seed germination (Dunand, 1992). Hence, this suggests the importance of selecting genotypes with sufficient mesocotyl elongation under submerged conditions when selecting recipients from semi-dwarf genotypes for higher % emergence and initial seedling vigor for weed management in DSR.

### Effects of Weed Growth on Shoot Biomass of Rice

The interaction effect of FD × rice genotype and weed level (*P*< 0.05) significantly influenced the rice shoot biomass production. The shoot biomass of tolerant genotype Khao Hlan On was not affected by weed competition under saturated and flooded conditions (Figure 10). However, IR64+AG1 and IR64 showed a reduction in their shoot biomass under saturated conditions and 2 cm FD due to weed competition, in addition to the flooding effect. Khao Hlan On, IR64+AG1, and IR64 had shoot biomass of 43.28, 41.55, and 40.38 g pot^−1^, respectively, under saturated weed-free conditions. IR64+AG1 and IR64 performed similarly in responding to weed competition under saturated conditions, for which both genotypes showed a 26% reduction in shoot biomass when sown with weeds. At 2 cm FD, IR64+AG1 produced 33.95 g shoot biomass pot^−1^ under weed-free conditions, and this declined by 10% under weedy conditions (*P*< 0.05). The moderately tolerant genotype IR64 showed a 14% reduction in shoot biomass due to weed competition under 2 cm FD compared with that of plants under weed-free conditions and at 2 cm FD. Khao Hlan On showed only a 13–15% shoot biomass reduction due to flooding compared with that in saturated conditions. The shoot biomass reduction of the rice genotypes due to weed competition was not significant under 5 cm FD (*P*> 0.05). However, shoot biomass reduction due to flooding was 15%, 27%, and 52% in Khao Hlan On, IR64+AG1, and IR64, respectively.

Biomass accumulation is a key determinant of weed competitiveness as it reflects resource capture under interference (Jabran and Chauhan, 2015). The tolerant genotype Khao Hlan On showed a higher and faster biomass accumulation under all conditions without a significant reduction due to weed competition. High anaerobic tolerance (Septiningsih et al., 2013a), fast coleoptile elongation, and higher initial seedling vigor (Ella et al., 2010) could be related to weed competitiveness under flooded conditions. Similarly, the taller shoots of this landrace (Kretzschmar et al., 2015) could also be one of the causes of the higher weed competitiveness of Khao Hlan On under saturated conditions. The relatively slow vegetative growth of IR64 (Fagi and Kartaatmadja, 2002), low initial seedling vigor, and delay in emergence above the water surface may have contributed to the low shoot biomass accumulation of IR64+AG1 under flooding+weedy conditions and also under saturated conditions.

### Effect of Weed Competition on Rice Leaf Area Production

**FIGURE 11:**
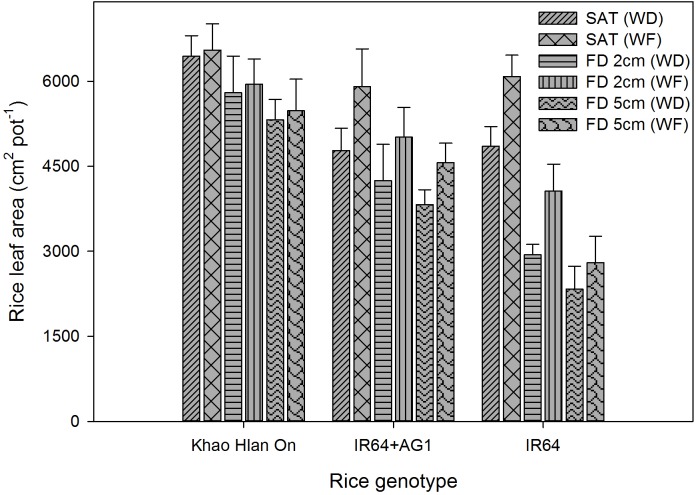
Genotypic variations in leaf area as affected by flooding and weed competition at 42 DAS. The vertical bars represent standard error of the mean. SAT, saturated; FD2cm, 2 cm flooding depth; FD5cm, 5 cm flooding depth; WD, weedy; WF, weed-free.

The three-way interaction of FD × weed competition × genotype was significant, and all influenced rice leaf area production at 42 DAS (Figure 11). Flooding reduced the leaf area production of all rice genotypes at different magnitudes depending on the tolerance (*P*< 0.05). The highest leaf area production was recorded under saturated weed-free conditions as 6,549, 5,901, and 6,084 cm^2^ pot^−1^ for Khao Hlan On, IR64+AG1, and IR64, respectively. At 2 cm and 5 cm FD, the leaf area of Khao Hlan On, IR64+AG1, and IR64 decreased by 11–16%, 15–22%, and 33–54%, respectively, compared to their growth under saturated weed-free conditions. The leaf area production of Khao Hlan On was not significantly affected by weed competition under both saturated and flooded conditions (*P*> 0.05). However, IR64+AG1 showed a 19%, 14%, and 15% reduction in leaf area due to weed competition under saturated conditions and 2 cm and 5 cm FD, respectively. Weed competition also reduced the leaf area of IR64 by 20%, 27%, and 16% under saturated conditions and 2 cm and 5 cm FD.

Taller plants with larger leaf area coverage will have higher competitiveness against weeds (Pérez de Vida et al., 2006) through shading (Chamara et al., 2016). The higher weed competitiveness of Khao Hlan On could be related to its inherent traits of appropriate height, initial seedling vigor, fast coleoptile elongation, and higher anaerobic tolerance (Ella et al., 2010). Flooding itself suppressed weed emergence and biomass accumulation to a greater extent at 42 DAS. However, IR64+AG1 still produced relatively lower leaf area in responding to weed competition, which could be due to the comparatively low initial seedling vigor, slow vegetative growth, and delay in emergence compared with those of Khao Hlan On. Therefore, the results highlighted further need to improve AG tolerance of IR64+AG1 to flooding through pyramiding of more QTLs for AG tolerance, early vigor, and larger leaf area, which would enhance the impact of early flooding on weed suppression and crop growth.

## Conclusion

The 1 cm SD under flooding resulted in better growth of all genotypes. The effect of flooding on crop growth is less when the crop is grown under shallow SD. However, SD greater than 1 cm could adversely affect crop growth and development. Shallow sowing, including 0.5 cm and surface seeding, is not recommended as it could adversely affect the strength of the vertical stand of rice, resulting in floating of seedlings. Therefore, 1 cm SD is recommended to facilitate better crop growth under flooded conditions. Selecting genotypes with sufficient mesocotyl elongation under submerged conditions could be important when selecting recipient parents from semi-dwarf genotypes for the introgression of AG traits to enhance emergence and initial seedling vigor for better weed management in DSR.

The 2 cm FD is sufficient to control problematic weed species to a greater extent. However, using flooding for weed management might also encourage the establishment of flood-tolerant weed species and other aquatic plants. The results of this study suggest a possible shift in populations of the currently problematic weed species in DSR, and that a few flood-tolerant weed species might predominate. More studies on the germination biology of these tolerant weed species are necessary to help develop IWM strategies for direct-seeding systems using AG-tolerant rice varieties. An effective direct-seeding system that encourages good rice establishment and discourages weed growth will facilitate the adoption of direct seeding as an efficient and cheaper system for crop establishment in the future.

## Author Contributions

BSCm was involved in designing and conducting the experiments, data gathering, statistical analysis, interpretation of data, developing figures, and drafting the manuscript. BM, VK, AMI, EMS, and BSCu were involved in designing the experiments, supervision during the research, and critical revision of the manuscript. All authors approved the final version of the manuscript.

## Conflict of Interest Statement

The authors declare that the research was conducted in the absence of any commercial or financial relationships that could be construed as a potential conflict of interest.

## Supplementary Material

FIGURE S1 Schematic diagram of the experimental designs.

TABLE S1 The ANOVA table with Pr (>F) values for data on emergence and plant height of rice genotypes.

## Abbreviations

AG: anaerobic germination
DSR: dry-seeded rice
PTR: puddled transplanted rice
FD: flooding depth
SD: sowing depth
DAS: days after seeding
TSBM: total shoot biomass
IWM: integrated weed management
AGB: aboveground biomass.
