# Supplementary figures and images for: Optimizing Sowing and Flooding Depth for Anaerobic Germination-Tolerant Genotypes to Enhance Crop Establishment, Early Growth, and Weed Management in Dry-Seeded Rice (Oryza sativa L.)
Source: Front Plant Sci. 2018 Nov 23;9:1654. doi: 10.3389/fpls.2018.01654 (PMC6265439; doi:10.3389/fpls.2018.01654)

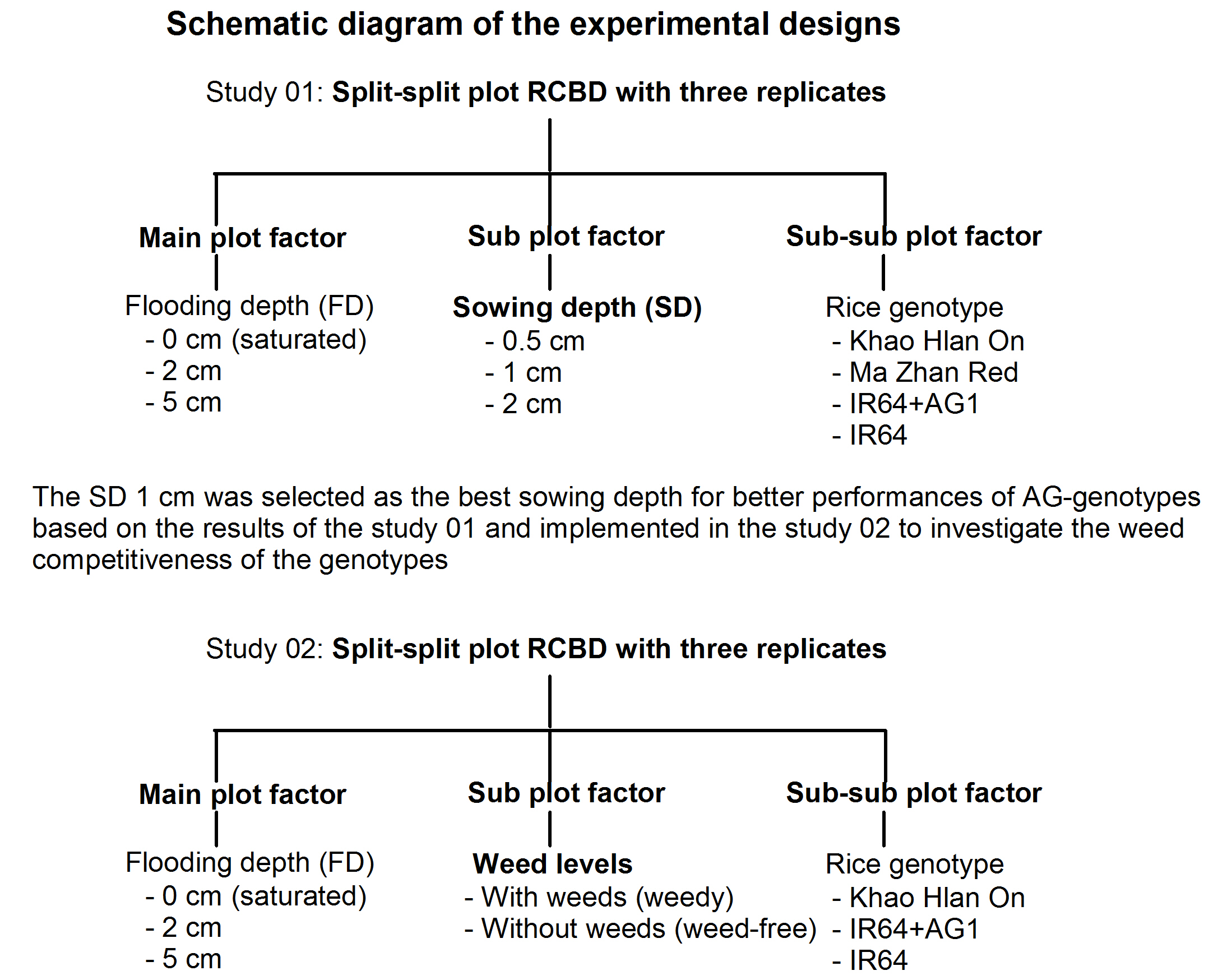

Supplement: FIGURE S1 — Schematic diagram of the experimental designs. [file Image_1.JPEG]
