# Supplementary material for: Optimizing Sowing and Flooding Depth for Anaerobic Germination-Tolerant Genotypes to Enhance Crop Establishment, Early Growth, and Weed Management in Dry-Seeded Rice (Oryza sativa L.)
Source: Front Plant Sci. 2018 Nov 23;9:1654. doi: 10.3389/fpls.2018.01654 (PMC6265439; doi:10.3389/fpls.2018.01654)
Supplement: TABLE S1 — The ANOVA table with Pr (>F) values for data on emergence and plant height of rice genotypes. [file Table_1.DOCX]

| Source of variation | df |  |  |  | Pr (> F) values for the tested parameter | | | | | |  |
| --- | --- | --- | --- | --- | --- | --- | --- | --- | --- | --- | --- |
|  |  | Rice seedling emergence at 14 DAS | Rice seedling emergence at 21 DAS | Rice seedling emergence at 28 DAS | Rice seedling emergence at 35 DAS | Plant height at 7 DAS | Plant height at 14 DAS | Plant height at 21 DAS | Plant height at 28 DAS | Plant height at 35 DAS | Plant height at 42 DAS |
| FD | 2 | <0.001 | <0.001 | <0.001 | <0.001 | <0.001 | 0.001 | 0.003 | 0.016 | 0.046 | 0.099 |
| SD | 2 | <0.001 | <0.001 | <0.001 | <0.001 | <0.001 | <0.001 | <0.001 | <0.001 | 0.001 | 0.005 |
| FD.SD | 4 | <0.001 | <0.001 | <0.001 | <0.001 | <0.001 | <0.001 | <0.001 | 0.014 | 0.030 | 0.161 |
| CV | 3 | <0.001 | <0.001 | <0.001 | <0.001 | <0.001 | <0.001 | <0.001 | <0.001 | <0.001 | <0.001 |
| FD.CV | 6 | <0.001 | <0.001 | <0.001 | <0.001 | <0.001 | 0.021 | <0.001 | 0.041 | 0.021 | 0.245 |
| SD.CV | 6 | <0.001 | <0.001 | <0.001 | <0.001 | <0.001 | 0.005 | <0.001 | 0.032 | 0.048 | 0.024 |
| FD.SD.CV | 12 | <0.001 | <0.001 | 0.014 | 0.022 | <0.001 | 0.022 | 0.003 | 0.081 | 0.277 | 0.289 |
